# Icariside II protects from marrow adipose tissue (MAT) expansion in estrogen-deficient mice by targeting S100A16

**DOI:** 10.1530/JME-24-0020

**Published:** 2024-09-18

**Authors:** Dong Li, Chenhao Cao, Zhuofan Li, Zhiyong Chang, Ping Cai, Chenxi Zhou, Jun Liu, Kaihua Li, Bin Du

**Affiliations:** 1 Department of Orthopedics, The Affiliated Hospital of Nanjing University of Chinese Medicine, Jiangsu Province Hospital of Chinese Medicine, Nanjing, Jiangsu, China

**Keywords:** Herba epimedii, Icariside II, marrow adipose tissue (MAT), osteoporosis, S100 calcium-binding protein A16 (S100A16)

## Abstract

Icariside II, a flavonoid glycoside, is the main component found *invivo* after the administration of Herba epimedii and has shown some pharmacological effects, such as prevention of osteoporosis and enhancement of immunity. Increased levels of marrow adipose tissue are associated with osteoporosis. S100 calcium-binding protein A16 (S100A16) promotes the differentiation of bone marrow mesenchymal stem cells (BMSCs) into adipocytes. This study aimed to confirm the anti-lipidogenesis effect of Icariside II in the bone marrow by inhibiting S100A16 expression. We used ovariectomy (OVX) and BMSC models. The results showed that Icariside II reduced bone marrow fat content and inhibited BMSCs adipogenic differentiation and S100A16 expression, which correlated with lipogenesis. Overexpression of S100A16 eliminated the inhibitory effect of Icariside II on lipid formation. β-catenin participated in the regulation adipogenesis mediated by Icariside II/S100A16 in the bone. In conclusion, Icariside II protects against OVX-induced bone marrow adipogenesis by downregulating S100A16, in which β-catenin might also be involved.

## Introduction

Icariside II, a flavonoid glycoside, is the major active ingredient of Herba epimedii. Herba epimedii is a traditional Chinese medicinal herb that has been used as an anti-rheumatic and anti-osteoporotic agent in China for thousands of years (Xia *et al*. 2010, Hong *et al*. 2022, He *et al*. 2023). Icariside II is suggested to possess anti-osteoporotic effects that play important roles in maintaining bone development and homeostasis (Luo *et al*. 2015, Liu *et al*. 2017, Szabó *et al*. 2022). Reports have demonstrated that Icariside II enhances the osteogenic differentiation of bone marrow mesenchymal stem cells (BMSCs) and prevents ovariectomy (OVX)-induced bone loss (Ming *et al*. 2013, Liu *et al*. 2017, Ma *et al*. 2022, Jiang *et al*. 2023). However, the mechanism by which Icariside II acts as an anti-osteoporotic factor remains to be elucidated.

Osteoporosis, a chronic metabolic systemic disease, is characterized by the progressive loss of bone mass and degeneration of bone tissue microarchitecture. Osteoporosis is considerably correlated with marrow adipose tissue (MAT) (Matsushita *et al*. 2022, Beekman *et al*. 2023). MAT, located in the femur, medullary canal of the tibia, and humerus, have been observed histologically for decades. The presence of adipocytes in mammalian bone marrow is unique, and adipocytes and bone cells are in close juxtaposition within the bone marrow. In many model systems, increased MAT has been associated with decreased bone mass and osteoporosis (Li *et al*. 2019, Sebo *et al*. 2019). This correlation often indicates that marrow adipocytes are negative regulators of bone mass. Marrow adipocytes partly arise from the BMSCs within the bone marrow. BMSCs are undifferentiated multipotent cells of mesenchymal origin with a self-renewal capacity and have the potential to differentiate into adipocytes and osteoblasts when exposed to specific growth stimuli. Although the association between marrow adipocytes and osteoporosis is clear, the mechanism of action remains unclear. One possible explanation for this is that BMSCs, but not osteoblasts, are stimulated to differentiate into adipocytes. The differentiation of BMSCs into adipocytes is influenced by many factors (Pierce *et al*. 2019, Qadir *et al*. 2020, Zhang *et al*. 2021, Gao *et al*. 2022).

S100 calcium-binding protein A16 (S100A16) promotes the differentiation of BMSCs into adipocytes and inhibits osteogenic differentiation (Li *et al*. 2013, Zhang *et al*. 2014). S100A16 is one of the most recently discovered members of the S100 family of proteins and is the largest subfamily of calcium-binding proteins. The S100 family comprises 22 components that act as extracellular factors and intracellular Ca^2+^ sensors that regulate cellular responses (Medkova *et al*. 2018, Li *et al*. 2023). However, the mechanisms by which S100A16 is involved in BMSC differentiation remain unclear. In our preliminary experiments, Icariside II inhibited S100A16 expression.

This study investigated the effects of Icariside II on adipocyte formation in the bone and the underlying mechanisms by which Icariside II inhibits S100A16 expression. This study aimed to lay the foundation for the application of Icariside II in osteoporosis.

## Materials and methods

### Establishment of an OVX model in mice

Six-week-old female wild-type C57BL/6J mice were obtained from Charies River. S100A16 knockout and transgenic mice were obtained from the Model Animal Research Center of Nanjing University (No. (2009) T67). All mice were housed under standard conditions (a temperature-controlled facility on a 12 h light–darkness cycle with free access to standard mouse food and water) in an specific pathogen free (SPF) environment at the Animal Core Facility of Nanjing Medical University. The experimental protocols listed below were reviewed and approved by the Animal Ethics and Welfare Committee (no. IACUC-1812002). Mice were anesthetized with 2.5% and 1% isoflurane (RWD, Nanjing, China) using an anesthesia machine. Dorsal hair was removed, and the mice were fixed in the prone position on a sterile animal operating table. Both ovaries were removed, sutured, and re-sterilized through the bilateral dorsal entrances. The surgical procedure and treatment in the sham-operated group were identical to those in the OVX group; however, the ovaries were not removed. A small animal warming blanket was used postoperatively until the patient awoke. Mice were numbered in order weighed and randomly assigned to four groups: sham, OVX, OVX + estrogen, and OVX + Icariside II (CAS No A0637, Chengdu Must Bio-Technology Co. Ltd, Chengdu, China) (*n* = 5). After 1 week, the mice were administered (intragastric (i.g.)) Icariside II (100 mg/kg/day, Guiechem, Shanghai, China, A0637) or estrogen (1 mg/kg/day, 17β-estradiol, Sigma, #E2758) twice a week for 18 weeks, whereas the sham and OVX group mice were administered CMC-Na (5 mg/kg/day). Body weight was recorded.

### Micro-CT scanning

After 18 weeks, the mice were anesthetized with 2.5% and 1% isoflurane (RWD) using an anesthesia machine, and micro-CT scanning of the femurs was performed using a SkyScan 1176 (Bruker microCT). The scanner was set at a voltage of 70 kV, a current of 200 μA, and a voxel size of 10 μm for analysis of the mouse femurs. After reconstruction of the scanned data, the regions of interest were selected for analysis of the bone and MAT.

### HE staining of femurs

After micro-CT scanning, the mice were sacrificed and femurs were collected, fixed in 4% paraformaldehyde, decalcified by shaking with 0.5 M ethylenediaminetetraacetic acid (EDTA), and then embedded in paraffin. The sections were 10 μm thick, dewaxed and rehydrated, stained using an HE kit, transparent in xylene, and sealed with a neutral resin.

### Measurement of total cholesterol (T-CHO) and triglyceride (TG)

At the end of experiment, the mice were anesthetized and blood was collected into tubes precoated with potassium-EDTA and centrifuged at 3000 ***g*** and 4°C for plasma preparation. The plasma levels of TG and T-CHO were determined using a Triglyceride Assay Kit (Beyotime S0219S) and a Total Cholesterol Assay Kit (Beyotime S0211S) according to the manufacturer’s instructions.

### Isolation and identification of BMSCs in mice

C57BL/6 female mice and other mice were obtained from the Animal Core Facility of Nanjing Medical University and Charies River. After cervical dislocation, mice were immersed in 75% alcohol for 5 min. The bilateral femurs were separated, and the adherent soft tissues were thoroughly removed. The epiphyses were removed by shearing, and marrow cells were harvested from the diaphyses by insertion of a syringe 10 mL needle. The bone marrow was repeatedly flushed with α-MEM using a needle. The cells were harvested and centrifuged (1006 ***g***, 5 min) to obtain cell precipitates. Supernatant was removed, and the pellet was suspended and cultured in a 10 cm dish with α-minimum essential medium (α-MEM) containing 10% fetal bovine serum (FBS, Gibco, 10270-106), 1% penicillin/streptomycin (Gibco, 15140122), and incubated under humidified conditions at 37°C in a 5% CO_2_ (Thermo Fisher). The cells were washed with PBS after 24 h and cultured by refreshing the culture medium twice a week. When the cells reached 80–90% confluence, they were passaged using 0.25% trypsin containing EDTA or seeded into plates.

### Differentiation into adipocytes

BMSCs in passages three–five were used for the differentiation studies. To induce adipogenic differentiation, cells were seeded into six-well plates at a density of 10^5^ cells/well and allowed to grow for 2 days to reach confluence (designated as d0) and cultured with adipogenic induction medium containing 10% FBS, 0.5 mmol/L 3-isobutyl-1-methyxanthine (Sigma, 5879), 1 μg/mL porcine insulin, and 1 mmol/L dexamethasone (Sigma). After 48 h of incubation (d2), the medium was replaced with α-MEM containing 10% FBS and 1 μg/mL insulin. On day 4, the medium was refreshed to α-MEM containing 10% FBS, and the incubation was continued for 4 days with two changes of medium.

### Oil Red O staining

The cells were washed three times with PBS, fixed with 4% formaldehyde at room temperature for 60 min, washed with PBS, and air-dried. After fixation, the cells were stained with freshly filtered 60% Oil Red O working solution for 60 min at room temperature. The cells were washed with 70% ethanol to remove unbound dye, and red-stained lipid droplets were observed and photographed under a microscope.

### Cellular TG assay

The cellular TG content was determined using a Triglyceride Assay Kit (Beyotime S0219S) according to the manufacturer’s instructions. The cells were cultured, induced to differentiate into adipocytes, washed twice with PBS, scraped in PBS, sonicated to homogenize the suspension, and assayed for total TGs.

### Western blot analysis

The BMSCs were lyzed using radioimmunoprecipitation assay lysis buffer (89901; Thermo Scientific). Protein extracts were separated on 6–12.5% sodium dodecyl sulfate polyacrylamide gel and transferred to nitrocellulose membranes, which were blocked with blocking buffer (5% dry milk) in tris-buffered saline with 0.1% Tween 20 for 1 h at room temperature and incubated with primary antibodies diluted in blocking buffer overnight at 4°C. The membranes were incubated with the corresponding secondary antibodies, and hybrid HRP was detected using Pierce ECL Western Blotting Substrate (Thermo Fisher Scientific). The relative expression levels of the target proteins were quantified using the ImageJ software (version 1.48).

### Realtime quantitative polymerase chain reaction (Q-PCR)

Total mRNA was extracted from the samples using the RNA easy Isolation Reagent (Vazyme, R701-01) following the manufacturer’s protocol, and cDNAs were synthesized using HiScript III RT SuperMix for qPCR (Vazyme, R323-01). Real-time Q-PCR was performed on a QuantStudio 7 (Applied Biosystems) using AceQ qPCR SYBR Green Master Mix (High ROX Premixed) (Vazyme, Q141-02). The mRNA expression of the target genes was detected and β-actin was used as an endogenous control. The expression of the target genes was presented relative to the control and data were analyzed using the 2^−Δ∆CT^ method. All primer pairs used are listed in Table S1.

### Immunofluorescence

BMSCs from 4-week-old rats were fixed in 4% PFA for 30 min at room temperature. After washing with PBS, the cells were permeabilized with 0.2% Triton X-100 and blocked with 5% BSA for 60 min. Samples were incubated with S100A16 antibodies and β-catenin antibodies overnight at 4°C. The next day, the samples were washed and incubated with goat anti-rabbit Alexa Fluor 594 (33112ES60, YEASEN) and goat anti-mouse Alexa Fluor 488 (33206ES60, YEASEN) secondary antibody for 1 h at room temperature and then counterstained with DAPI (D8417, Sigma-Aldrich). The images were captured using a fluorescence microscope (IX83; Olympus).

### Statistics

The exclusion criteria were not included in the analysis. All data are presented as mean ± s.e.m. When data between samples were normally distributed and had homogeneity of variance, paired Student’s *t*-test and one-way analysis of variance (ANOVA) corrected with Tukey's honest significant difference test were used to compare the statistical significance between two groups (using a two-tailed Student’s *t*-test) and among three or more groups (using one-way ANOVA). The Mann–Whitney–Wilcoxon test and Dunnett's *t*-test were used to compare the statistical significance between two or more groups. Differences were considered statistically significant at *P* < 0.05. Statistical significance analysis was performed using R4.04.

## Results

### Icariside II alleviates estrogen-deficiency-induced lipogenesis

**Figure 1:**
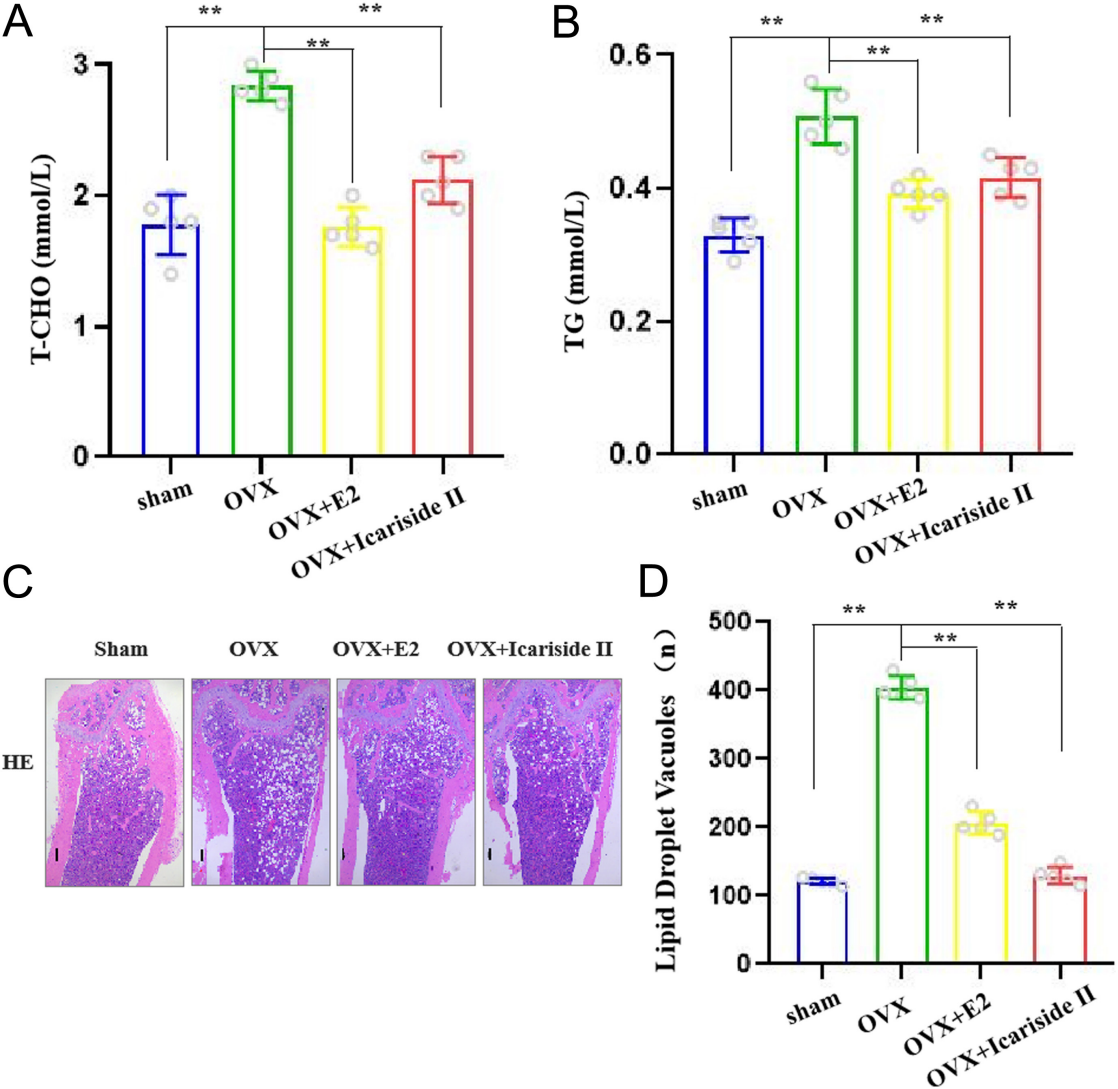
Icariside II reduces estrogen-deficiency-induced lipogenesis. (A) The plasma concentration of total cholesterol (T-CHO), *n* = 5/group. (B) The plasma concentration of triglyceride (TG), *n* = 5/group. (C) HE staining of femoral marrow cavity of sham, OVX, OVX + E2, and OVX + Icariside II groups, scale bar = 100 μm. (D) Quantitative results of C, *n* = 5/group. Each bar represents mean ± s.e.m. values. *P < 0.05 vs the control group; **P < 0.01 vs the control group. The E2 group is the positive control. A full-colour version of this figure is available at https://doi.org/10.1530/JME-24-0020.

To assess the effects of Icariside II on lipid synthesis, we used an OVX model. At the end of the experiment, the plasma levels of T-CHO and TGs significantly increased after OVX and decreased after the administration of Icanriside II and estrogen (E2) (positive control) (Fig. 1A and B). The effect of Icariside II on the femur after OVX was investigated using hematoxylin and eosin (HE) staining, which was used to analyze the bone microstructure and lipid droplets. The results showed that the number of adipocytes in the femoral marrow cavity of OVX mice was remarkably higher than that in sham mice. A distinct reduction in the number of droplets in the femoral marrow cavity after Icariside II and E2 administration was observed (Fig. 1C and D). The body weights of all the mice were monitored every week. Compared with the OVX group, Icariside II and E2 (positive controls) treatment reduced body weight gain (Supplementary Figure 1A, see the section on supplementary materials given at the end of this article). These results indicate that OVX leads to obesity and Icariside II reduces bone marrow fat content.

### Icariside II inhibited BMSC adipogenic differentiation

**Figure 2:**
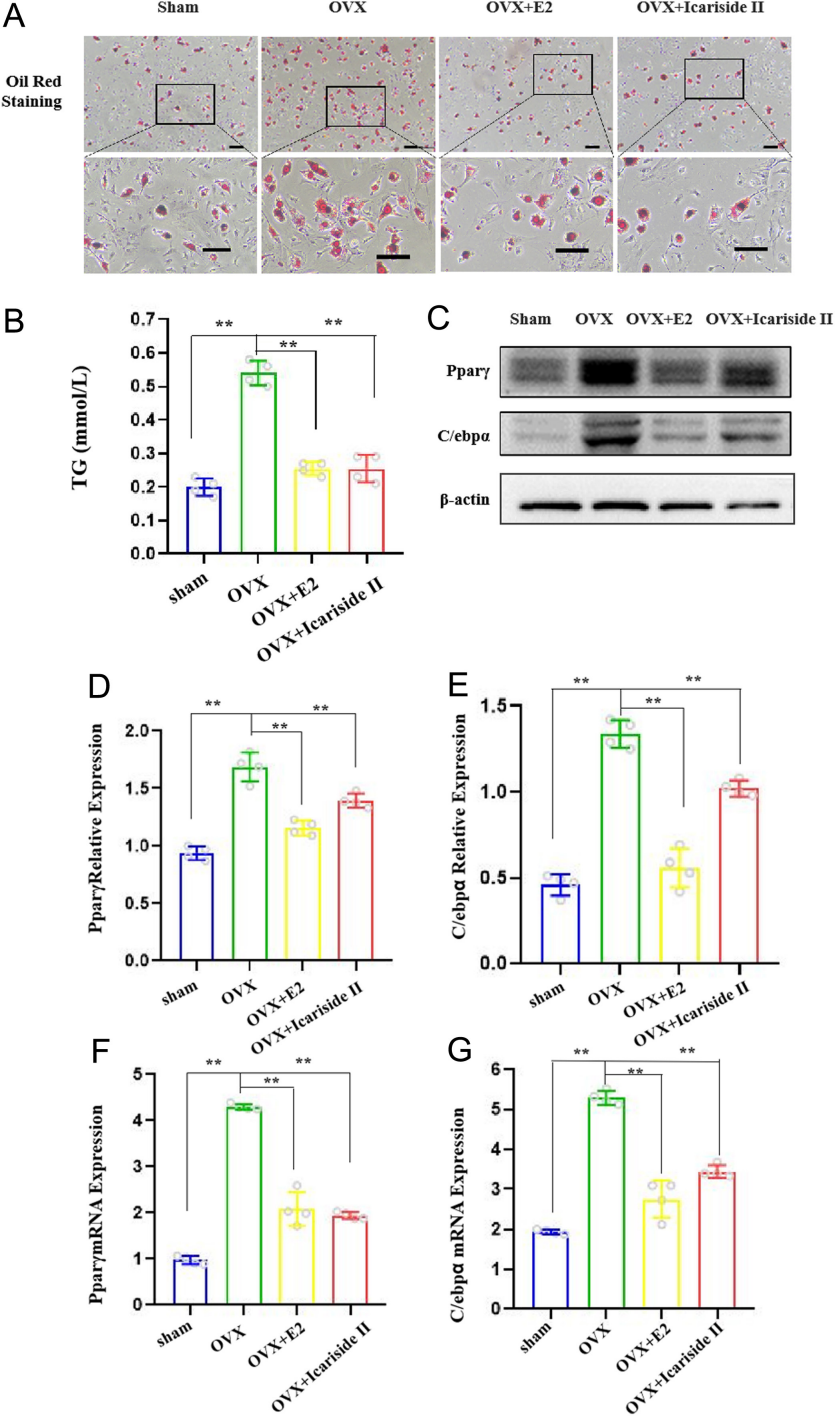
Icariside II influences the BMSC adipogenic differentiation. (A)Oil Red O staining of lipid droplets after adipogenic differentiation of BMSCs from sham, OVX, OVX + E2, and OVX + Icariside II groups, scale bar = 100 μm or 50 μm. (B)Quantitative determination of triglyceride accumulation in cells of A. (C)Western blot. (DandE) Scanning and densitometry of Pparγ and C/ebpα, *n* = 4/group. (F and G) Q-PCR assay of Pparγ and C/ebpα, *n* = 4/group. Each bar represents mean ± s.e.m. values. *P < 0.05 vs the control group; **P < 0.01 vs the control group. A full-colour version of this figure is available at https://doi.org/10.1530/JME-24-0020.

First, mouse BMSCs were identified. Primary BMSCs were acquired from the femoral bone marrow of C57BL/6J mice. Primary BMSCs exhibited stemness and multidirectional differentiation (Supplementary Figure 2). Adipocytes and osteoblasts share a common progenitor, BMSCs. Given the inhibition of MAT expansion observed in the Icariside II group, we wanted to determine whether the differentiation of BMSCs into adipocytes was affected by Icariside II. We isolated BMSCs from mice and stimulated them to differentiate into adipocytes. Oil Red O staining showed that the OVX group had a large number of red-stained fat particles deposited in the cells compared to the sham group. However, the fat droplets in the cells were significantly reduced after treatment with Icariside II and estrogen (Fig. 2A). Consistent with these staining patterns, quantitative analysis of cellular triglycerides (TG) showed that TG accumulation was significantly higher in cells from the OVX group but lower in cells treated with Icariside II and estrogen (positive control) (Fig. 2B), suggesting that Icariside II inhibits BMSC differentiation into adipocytes. To confirm the functional role of Icariside II, we examined the expression levels of adipocyte marker genes.

The relative expressions of adipogenic genes (Pparγ and C/ebpα) were increased in BMCs of OVX mice and decreased in Icariside II and estrogen group (Fig. 2C–G). The cells induced by icariin II were difficult to differentiate into adipocytes as determined by decreased Oil Red O staining of fat droplets and decreased expression of adipogenic markers, including Pparγ and C/ebpα. To assess the mechanism of impaired adipogenesis generated by Icariside II, we employed an S100A16 genetically modified mouse model in the present study.

### Icariside II inhibited S100A16 expression, which correlated with lipogenesis

**Figure 3:**
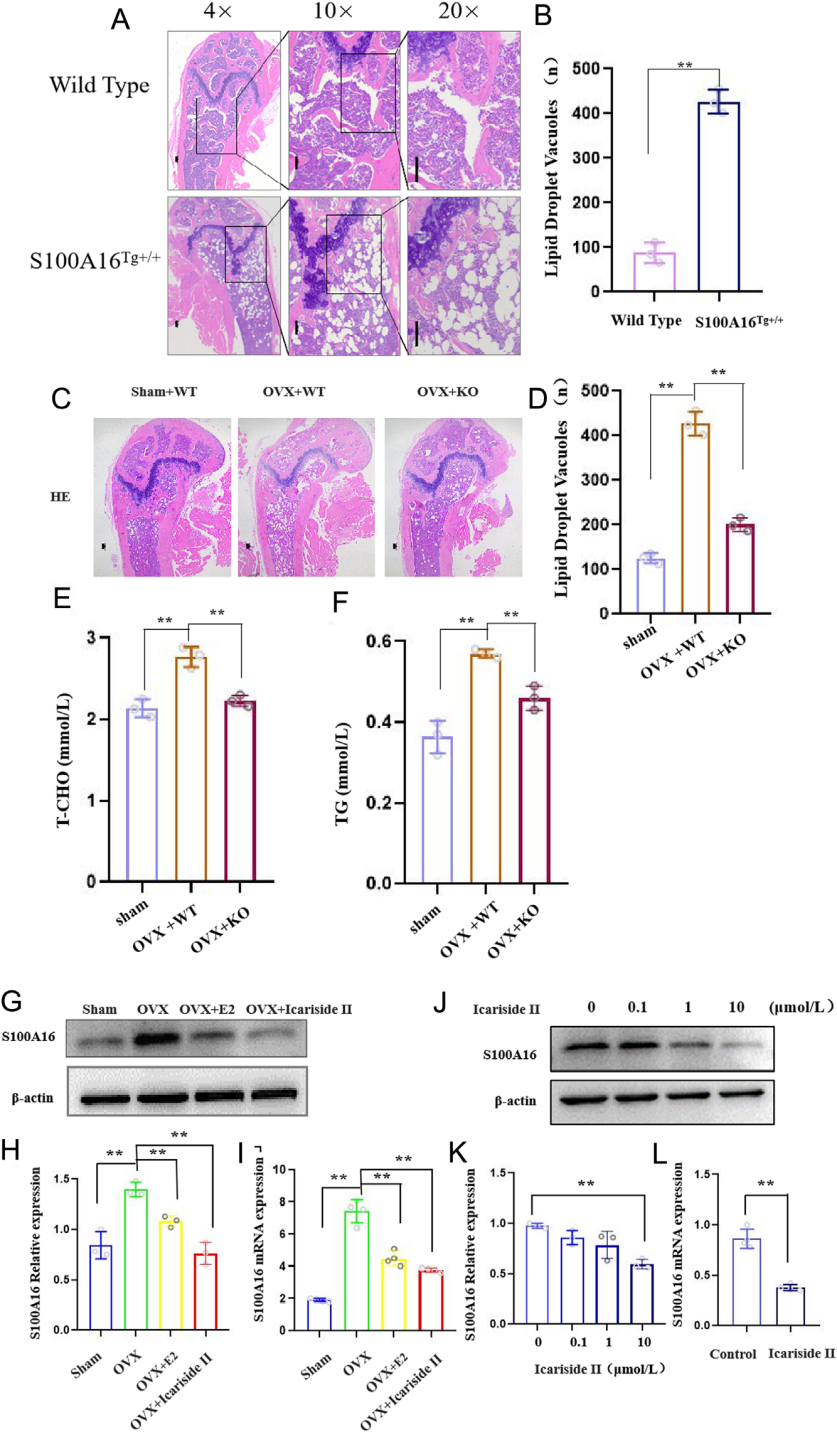
Icariside II inhibited S100A16 expression. (A) HE staining of femoral marrow cavity from S100A16^TG+/+^, scale bar = 100 μm or 50 μm. (B)Quantitative results of A. (C)HE staining of femoral marrow cavity from S100A16^KO+/−^, scale bar = 100 μm. (D) Quantitative results of C. (E) The plasma concentration of T-CHO, *n* = 3/group. (F) The plasma concentration of TG, *n* = 3/group. (G and H) Western blot and scanning densitometry of S100A16 from sham, OVX, OVX + E2, and OVX + Icariside II groups, *n* = 3/group. (I) Q-PCR assay of S100A16 from sham, OVX, OVX + E2, and OVX + Icariside II groups, *n* = 4/group. (J and K) Western blot and scanning densitometry of S100A16 from Icariside II-treated groups (0, 0.1, 1, and 10 μmol/L), *n* = 3/group. (L) Q-PCR assay of S100A16 from Icariside II-treated groups (10 μmol/L), *n* = 4/group. Each bar represents mean ± s.e.m. values. *P < 0.05 vs the control group; **P < 0.01 vs the control group. A full-colour version of this figure is available at https://doi.org/10.1530/JME-24-0020.

Previous studies have shown that S100A16 promotes visceral adipogenesis (Liu *et al*. 2011). To understand the functional significance of S100A16 in bone marrow fat, we assessed fat droplets in the bones of S100A16 genetically modified mice. S100A16 overexpression increased the number of lipid droplets in MAT (Fig. 3A and B). S100A16 deficiency protected mice from an ovariectomy-induced increase in lipid droplets (Fig. 3C and D). The concentrations of TG and T-CHO were elevated but decreased by the downregulation of S100A16 (Fig. 3E and F). These data suggest that overexpression of S100A16 was sufficient to stimulate the expansion of lipid droplets in MAT in femurs and that downregulation of S100A16 likely contributed significantly to the inhibition of MAT expansion induced by ovariectomy.

Does Icariside II exert a regulatory effect on S100A16? To examine the effect of Icariside II on the expression of S100A16, we extracted protein and RNA from BMSCs isolated from the model shown in Fig. 1. S100A16 protein levels were determined by western blotting, and relative S100A16 mRNA levels were determined by Q-PCR. Western blotting showed that S100A16 expression was downregulated in the Icariside II-treated group compared to that in the OVX group. The Q-PCR analysis was consistent with the western blot results (Fig. 3G–I).

To further confirm this result, we isolated BMSCs from the bone marrow of C57BL/6J mice, treated them with different concentrations of Icariside II, and then western blot and Q-PCR were used to analyze the expression of S100A16. As expected, under Icariside II stimulation, the expression level of S100A16 decreased. The result showed that the expression of S100A16 was inhibited in the presence of Icariside II 1 and 10 μmol/L, compared to the untreated control group. The maximal inhibitory effect was achieved by the concentration of 10 μmol/L (Fig. 3J and K). Q-PCR analysis further confirmed these results (Fig. 3L). Therefore, we speculate that S100A16 could be a downstream effector through which Icariside II regulates MAT expansion.

### Overexpression of S100A16 can eliminate the inhibitory effect of Icariside II on lipid formation

**Figure 4:**
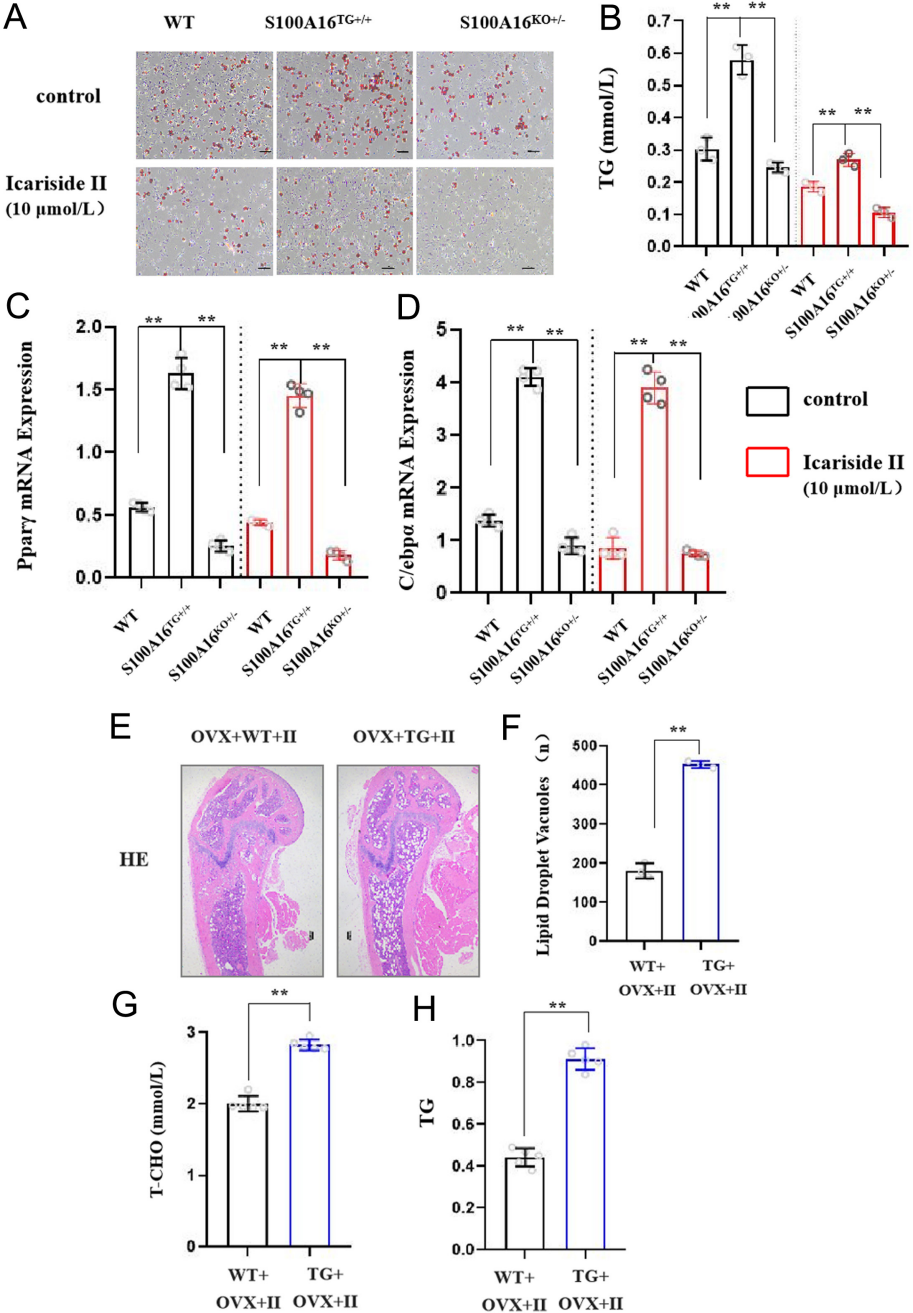
S100A16 balanced the effect of Icariside II. (A) Oil Red O staining of BMSC adipogenic differentiation from WT, S100A16^TG+/+^, and S100A16^KO+/−^ mice, scale bar = 100 μm. (B)Quantitative determination of triglyceride accumulation in cells of A.(CandD) Q-PCR assay of Pparγ and C/ebpα, *n* = 4/group. (E) HE staining of femoral marrow cavity from WT and S100A16^TG+/+^ mice treated with Icariside II, scale bar = 100 μm. (F) Quantitative results of E, *n* = 3/group. (G) The plasma concentration of T-CHO. (H) The plasma concentration of TG, *n* = 5/group. Each bar represents mean ± s.e.m. values. *P < 0.05 vs the control group; **P < 0.01 vs the control group. A full-colour version of this figure is available at https://doi.org/10.1530/JME-24-0020.

To obtain direct evidence that S100A16 influences the effect of Icariside II, we utilized BMSCs isolated from S100A16 transgenic and S100A16 knockdown mice (Zhang *et al*. 2022). Cells from wild-type, S100A16^TG+/+^, and S100A16^KO+/−^ mice were treated with Icariside II. Oil O Red staining revealed that Icariside II inhibited lipid droplet formation. However, overexpression of S100A16 led to a decrease in the inhibitory effect of Icariside II on lipid formation, and lipid formation increased. BMSCs with low S100A16 expression stimulated with Icariside II had a strong inhibitory effect on lipid formation, and fever lipid droplets accumulated in cells from S100A16^KO+/−^ mice (Fig. 4A). Quantitative determination of triglyceride accumulation is consistent with such staining patterns. Quantitative analysis of cellular TGs showed that TG accumulation was significantly higher in the cells from S100A16^TG+/+^ mice but lower in the cells from S100A16^KO+/−^ mice than the control cells (Fig. 4B). The genes associated with adipogenic differentiation (Pparγ and C/ebpα) were increased in the S100A16^TG+/+^ mice (Fig. 4C and D), while these genes were decreased in the S100A16^KO+/−^ mice compared with wild type. However, Icariside II did not affect the expression of these two genes. This may be caused by various factors such as the selection of differentiation time points.

Similar to the cell model, HE staining of the femur revealed a greater increase in bone marrow in S100A16 transgenic mice. Overexpression of S100A16 counteracted the inhibitory effect of Icariside II on lipid formation and increased adipogenesis (Fig. 4E andF), while those related to the concentrations of TG and T-CHO (Fig. 4G and H) showed the same results. These data indicated that S100A16 is critical to bone marrow adipogenesis in response to Icariside II.

### β-catenin may participate in regulating adipogenesis mediated by Icariside II/S100A16 in the bone

**Figure 5:**
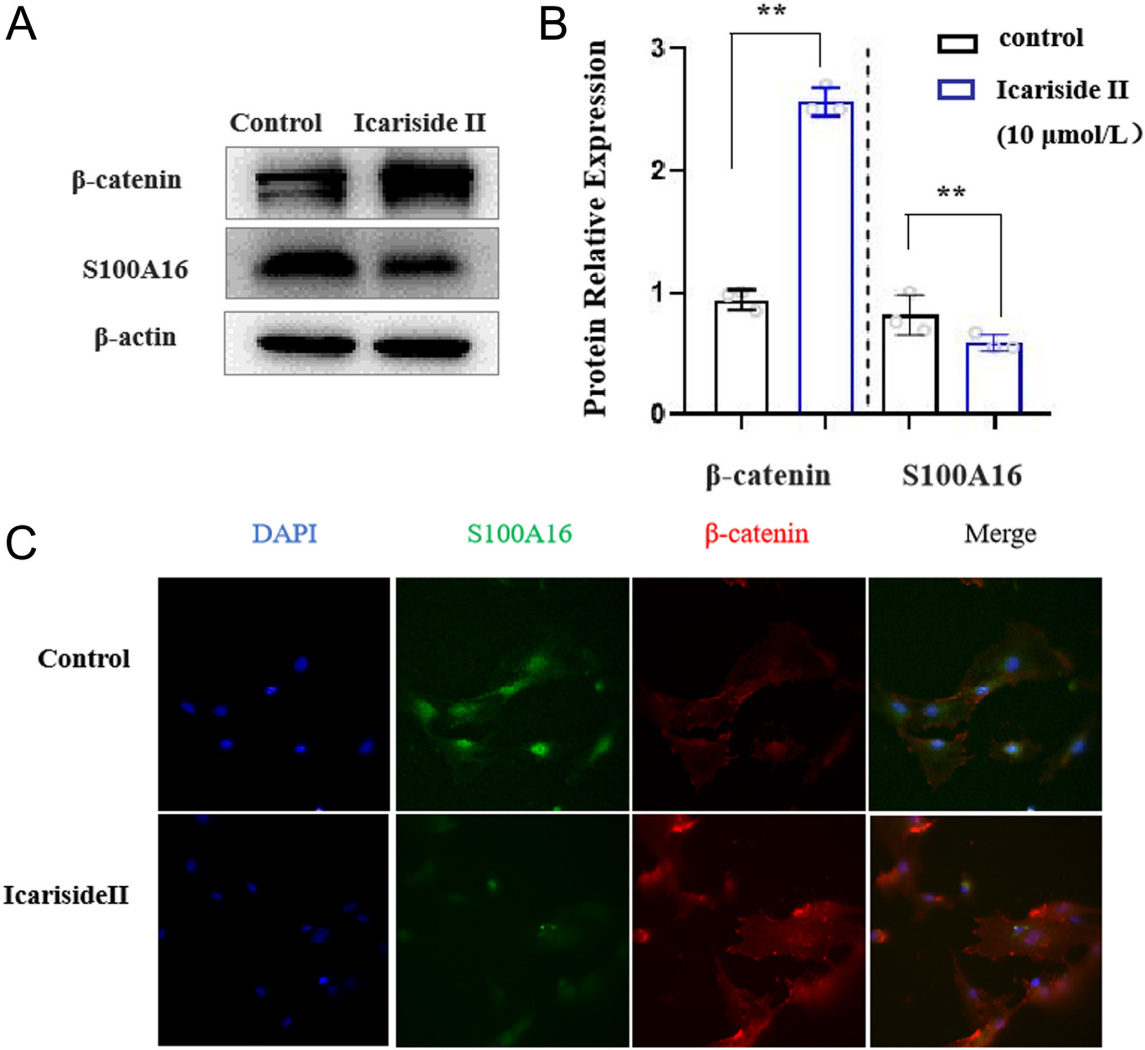
The effect of β-catenin in the progress of Icariside II/S100A16 in the bone. (A and B) Western blot and scanning densitometry of S100A16 and β-catenin in BMSCs treated with Icariside II (10 μmol/L), scale bar = 50 μm, *n* = 3/group. (C) Immunofluorescence images of S100A16 and β-catenin in BMSCs, *n* = 3/group. Each bar represents mean ± s.e.m. values. *P < 0.05 vs the control group; **P < 0.01 vs the control group. A full-colour version of this figure is available at https://doi.org/10.1530/JME-24-0020.

Thus, Icariside II may suppress bone marrow adipogenesis by inhibiting S100A16 expression. β-catenin pathway was strongly linked with bone formation, which has been demonstrated to promote BMSC osteogenic differentiation. Upregulated β-catenin is translocated to the nucleus and stimulates the expression of various downstream genes. We used western blot and immunofluorescence staining to detect β-catenin expression levels in BMSCs treated with Icariside II. When confluent, undifferentiated WT BMSCs were treated with 10 mM Icariside II, S100A16 was markedly downregulated, whereas β-catenin expression was upregulated compared to the control group (Fig. 5A and B). The immunofluorescence staining results for S100A16 are shown in Fig. 5C; we also observed the same results. Notably, decreased S100A16 expression and increased β-catenin expression were observed in BMSCs treated with Icariside II at the same time. Accordingly, we deduced that Icariside II inhibited BMSCs’ adipogenic differentiation potential by the downregulation of S100A16 in which β-catenin might be involved. However, its role and mechanisms remain unclear.

## Discussion

Bone marrow adipose tissue (BMAT) is a unique fat depot located within the skeleton and composed of large adipocytes. BMAT expansion is primarily attributed to aging and sex steroid deficiency, and BMAT plays an important role in the pathogenesis of bone loss in osteoporosis (Tencerova *et al*. 2021, Beekman *et al*. 2023, Guimarães *et al*. 2024). Increasing evidence suggests that one of the key mechanisms of estrogen deficiency-associated osteoporosis is the altered differentiation of BMSCs. BMSCs is a discrete group of preadipocyte-like cells in BMAT, possessing trilineage differentiation potential into chondrocytes, osteoblasts, and adipocytes (Zwick *et al*. 2018, Pachón-Peña & Bredella 2022, Li *et al*. 2023). We extracted BMSCs, induced their differentiation, and found that they were indeed able to successfully differentiate into chondrocytes, osteoblasts, and adipocytes. To determine the cell type, we used Q-PCR to detect the expression of various cell marker genes (Supplementary Figure 2). BMSCs can undergo multi-directional differentiation.

The expansion of BMAT is caused by an increase in adipocyte size and/or number, which may be a result of enhanced adipogenesis of BMSCs. Increased BMSC differentiation into adipocytes is one of the key mechanisms suggested to play a role in the expansion of BMAT and the decrease in bone mass, thereby leading to osteoporosis during aging. Many conditions are associated with the differentiation of BMSCs into adipocytes, including obesity, aging, osteoporosis, estrogen deficiency, anorexia, hyperlipidemia, and glucocorticoids (Gimble & Nuttall 2012, Rath *et al*. 2016, Li *et al*. 2018).

Icariside II is a major bioactive metabolite of Epimedii Herba, a traditional Oriental medicine that exhibits anti-osteoporotic activity. Although Icariside II has anti-osteoporotic effects, its pathway and mechanism of action remain unknown (Zhang *et al*. 2012, Liu *et al*. 2014, Arief *et al*. 2015, Zhao *et al*. 2016, Wang *et al*. 2018). In our study, we used an OVX model, which had the same effect as E2, which was efficacious at lowering body weight gain (Supplementary Figure 1). Administration of Icanriside II decreased the plasma levels of T-CHO and TG (Fig. 1A and B). Importantly, HE staining showed that Icariside II reduced the droplet size in the femoral marrow cavity (Fig. 1C and D). This suggests that Icariside II can reduce bone marrow fat content.

How does Icariside II exert its anti-lipogenic effect? BMSCs may be a key factor. In our study, we isolated BMSCs from the mice used in the animal experiments described above and stimulated them to differentiate into adipocytes. Oil Red O staining showed that the OVX group had a large number of red-stained fat particles deposited in the cells compared to the sham group. However, the fat droplets in the cells were significantly reduced after Icariside II and estrogen treatments (Fig. 2A and B). The relative expressions of adipogenic genes (Pparγ and C/ebpα) were increased in BMCs of OVX mice and decreased in Icariside II and estrogen group (Fig. 2C–G). This suggests that Icariside II may inhibit the differentiation of BMSCs into adipocytes in the bone, further acting as an anti-lipogenic agent. BMSC differentiation of BMSCs into adipocytes may contribute to osteoporosis.

Subsequently, we studied the mechanism by which Icariside II inhibited the differentiation of BMSCs into adipocytes. S100A16 is a small-molecule functional Ca^2+^-binding protein, which is expressed ubiquitously, and its mRNA levels have been detected in all human tissues (Medkova *et al*. 2018). In our early research, we revealed for the first time that the S100A16 protein is a novel adipogenesis-promoting factor and that increased expression of S100A16 in 3T3-L1 adipocytes can have a negative impact on insulin sensitivity (Liu *et al*. 2011, Zhang *et al*. 2012, Yuan *et al*. 2022). In the present study, we examined the effects of S100A16 on bone marrow adipocytes. Using S100A16 genetically modified mice, we found that overexpression of S100A16 increased lipid droplets in MAT, and S100A16 deficiency protected mice from OVX-induced lipid droplet increase (Fig. 3). The TG and T-CHO concentrations showed similar changes. These data suggest that S100A16 has a positive regulatory effect on the adipogenic differentiation of BMAT in the femur.

Both Icariside II and S100A16 have regulatory effects on lipid formation in BMAT. What are the regulatory mechanisms that exist between S100A16 and Icariside II? Our results showed that S100A16 expression was downregulated in the Icariside II-treated group compared to that in the OVX group. S100A16 may be a downstream effector through which Icariside II regulates MAT expansion. Using a BMSC cell model (isolated from S100A16 transgenic mice and S100A16 knockdown mice) and a mouse model (S100A16 transgenic mice), we confirmed that S100A16 may be critical for BM adipogenesis in response to Icariside II (Fig. 4).

Icariside II suppresses bone marrow adipogenesis by inhibiting S100A16 expression. Specific cellular molecules regulate this process. We focused on the β-catenin signaling molecule. β-catenin signaling pathway performs a function in the differentiation of BMSC into osteoblasts or adipocytes (Li *et al*. 2013, Shen *et al*. 2020, Zhao *et al*. 2020). Our results showed that Icariside II upregulated β-catenin expression (Fig. 5). Together, these data implied that Icariside II might increase β-catenin signaling to inhibit BMSCs toward adipocyte differentiation. In conclusion, this study investigated the protective effect of Icariside II against OVX-induced bone marrow adipogenesis based on the downregulation of S100A16, in which β-catenin might be involved.

## Supplementary Materials

Table S1 Primers used and their representative sequences

Supplementary Figure 1

Supplementary Figure 2

## Declaration of interest

The authors declare that there is no conflict of interest that could be perceived as prejudicing the impartiality of the study reported.

## Funding

This work was supported by grants from the National Natural Science Foundation of Chinahttp://dx.doi.org/10.13039/501100001809 (nos 81873105, 82374164, 82074471).

## Author Contribution statement

DL: conceptualization, project administration, supervision, methodology, writing and original draft. CC: formal analysis, methodology. ZL: investigation and data curation. ZC: investigation. PC, CZ, JL, and KL: data curation, investigation. BD: funding acquisition, writing, review, editing and supervision.
