# Supplementary material for: Icariside II protects from marrow adipose tissue (MAT) expansion in estrogen-deficient mice by targeting S100A16
Source: J Mol Endocrinol. 2024 Sep 18;73(3):e240020. doi: 10.1530/JME-24-0020 (PMC11466200; doi:10.1530/JME-24-0020)
Supplement: Table S1 Primers used and their representative sequences [file supplementary_table_1.pdf]

Table S1 Primers used and their representative sequences

Primers		Sequences
S100A16	Forward	CGGACACAGGGAACCGAAAG
	Reverse	GTCCAGTATTCGTCAAAGCAGA
β-actin	Forward	AGTGTGACGTTGACATCCGTA
	Reverse	GCCAGAGCAGTAATCTCCTTCT
SOX9	Forward	GAGCCGGATCTGAAGAGGGA
	Reverse	GCTTGACGTGTGGCTTGTTC
PPARγ	Forward	TCGCTGATGCACTGCCTATG
	Reverse	GAGAGGTCCACAGAGCTGATT
C/EBPα	Forward	CAAGAACAGCAACGAGTACCG
	Reverse	GTCACTGGTCAACTCCAGCAC
RUNX2	Forward	AACGATCTGAGATTTGTGGGC
	Reverse	CCTGCGTGGGATTTCTTGGTT
Bmp2	Forward	GGGACCCGCTGTCTTCTAGT
	Reverse	TCAACTCAAATTCGCTGAGGAC
Aggrecan	Forward	CCTGCTACTTCATCGACCCC
	Reverse	AGATGCTGTTGACTCGAACCT
cyclin D1	Forward	GCGTACCCTGACACCAATCTC
	Reverse	CTCCTCTTCGCACTTCTGCTC
COL-II	Forward	TGAAGACCCAGACTGCCTCAA
	Reverse	AGCCGCGAAGTTCTTTTCTCC
Myc	Forward	ATGCCCCTCAACGTGAACTTC
	Reverse	CGCAACATAGGATGGAGAGCA
CD44	Forward	TCTGCCATCTAGCACTAAGAGC
	Reverse	GTCTGGGTATTGAAAGGTGTAGC
