# Supplementary figures and images for: Icariside II protects from marrow adipose tissue (MAT) expansion in estrogen-deficient mice by targeting S100A16
Source: J Mol Endocrinol. 2024 Sep 18;73(3):e240020. doi: 10.1530/JME-24-0020 (PMC11466200; doi:10.1530/JME-24-0020)

Supplemental  
Fig.1A

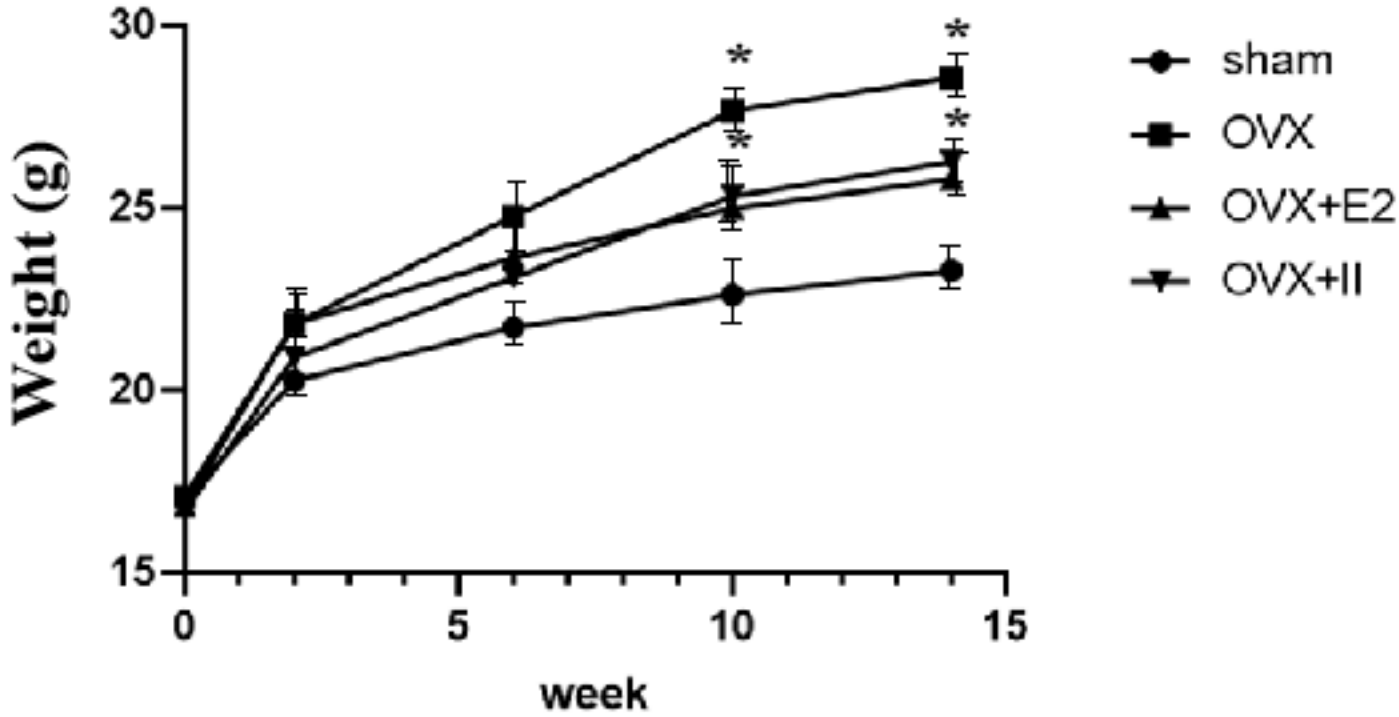

Supplement: Supplementary Figure 1 [file supplementary_figure_1.pdf]

Supplemental  
Fig.2A

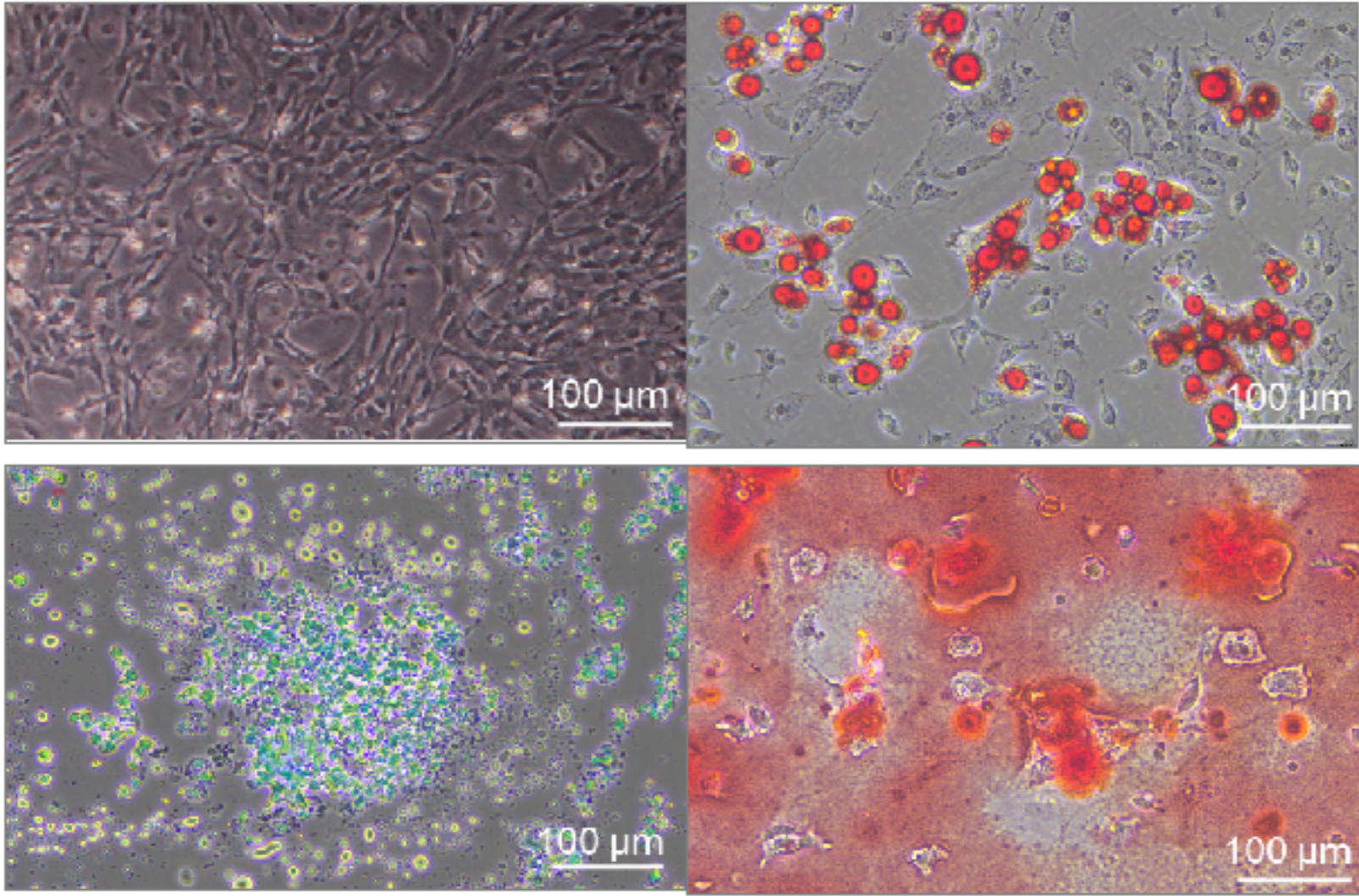

Fig.2B

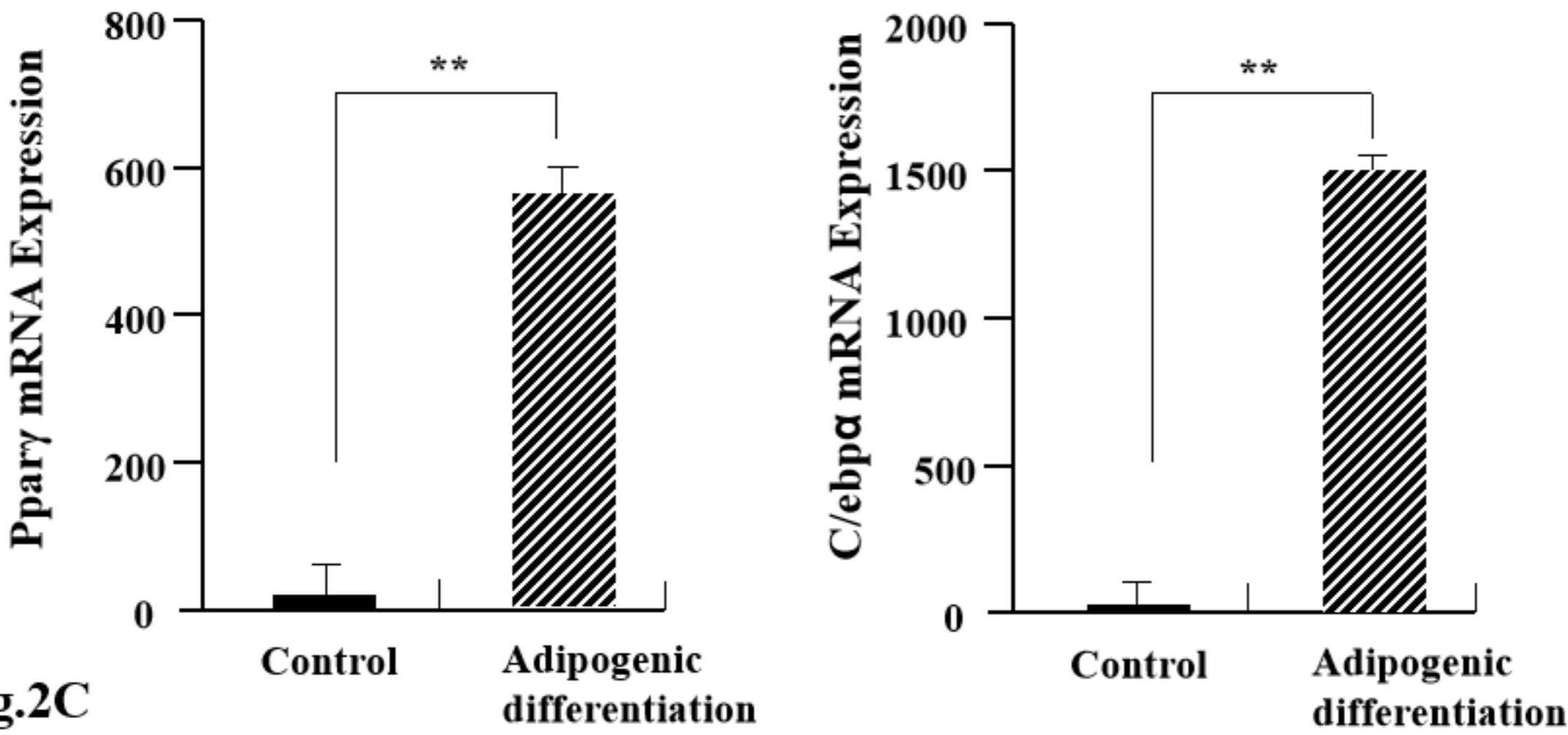

Fig.2C

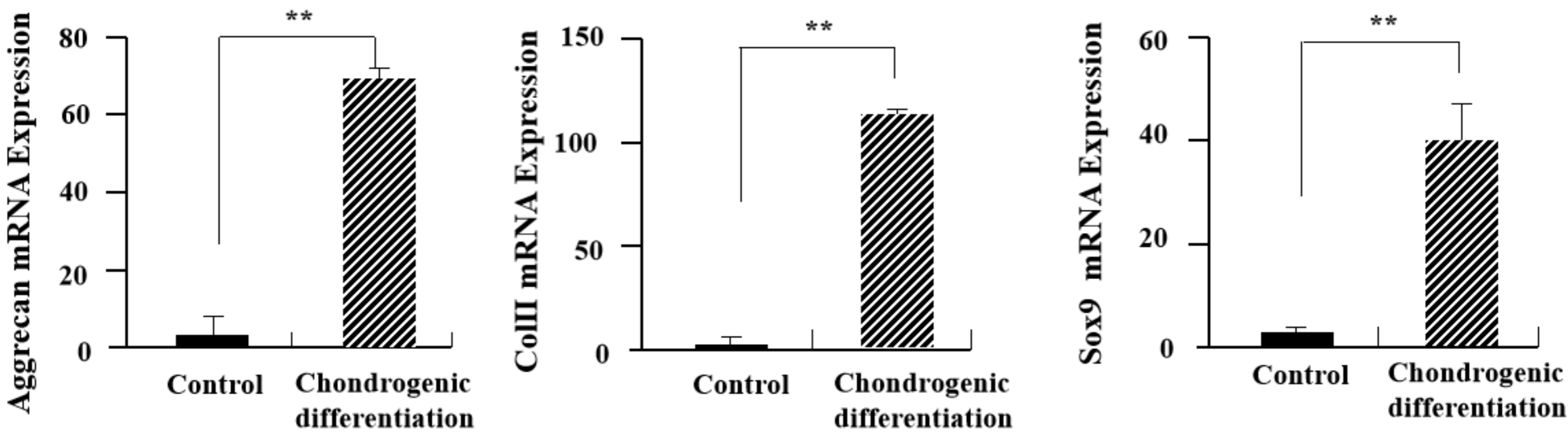

Fig.2D

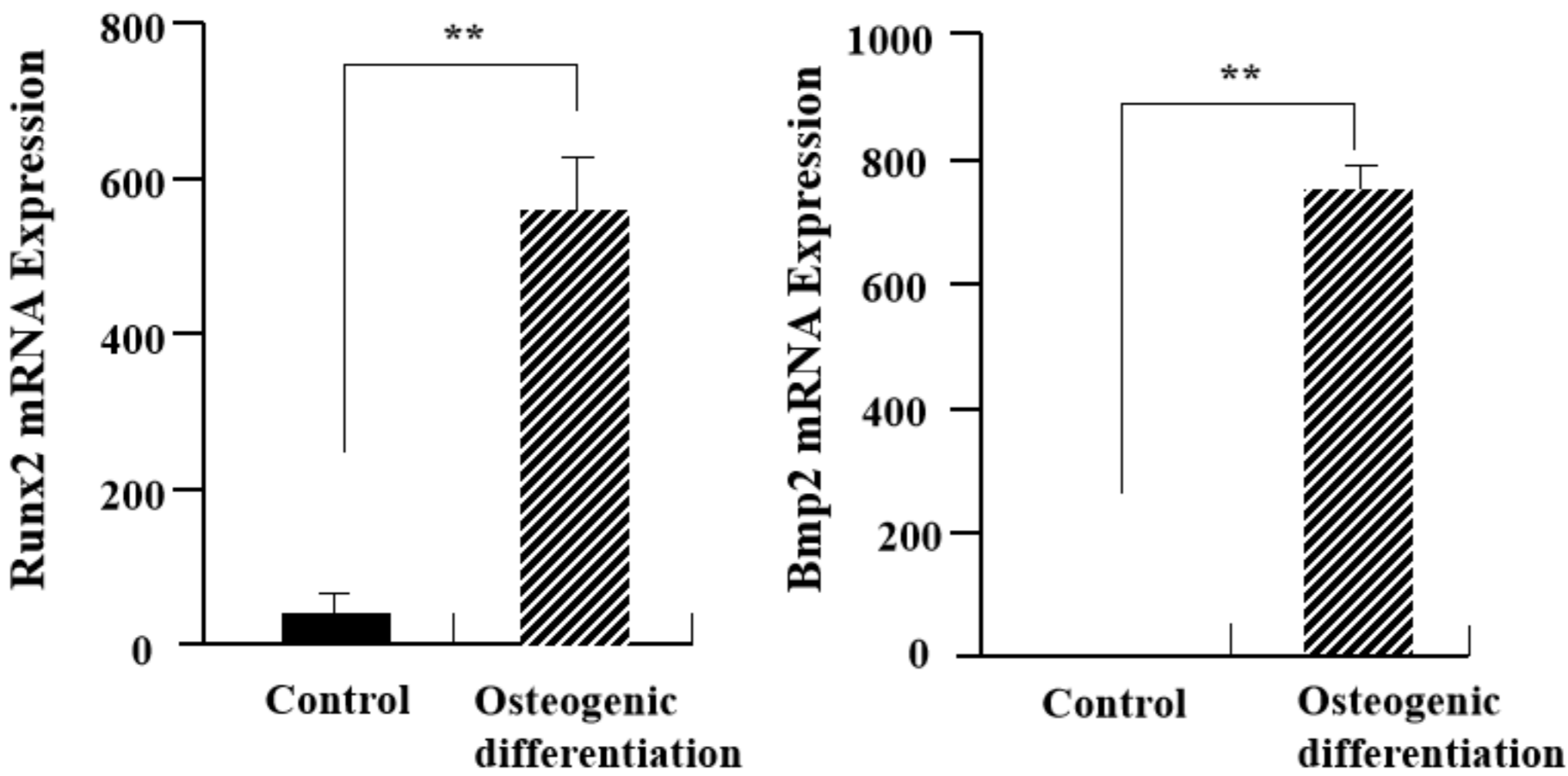

Supplement: Supplementary Figure 2 [file supplementary_figure_2.pdf]
